# Psychosocial factors and mental health among undergraduate expatriate students in the UAE: a moderated mediation model

**DOI:** 10.3389/fpsyg.2026.1858806

**Published:** 2026-10-05

**Authors:** Nida Fayyaz, Siti Aisyah Panatik

**Affiliations:** 1 Faculty of Social Science and Humanities, Universiti Teknologi Malaysia, Johor Bahru, Malaysia; 2 School of Human Resource Development and Psychology, Universiti Teknologi Malaysia, Johor Bahru, Malaysia

**Keywords:** ecological systems theory, expatriate students, mental health, moderated mediation, psychosocial factors, resilience, self-efficacy, UAE higher education

## Abstract

This conceptual article suggests a moderated mediation model that will be used to explore how psychosocial variables influence the positive mental health of undergraduate expatriate students in the United Arab Emirates. The article does not provide any empirical data, findings based on samples, or statistical testing, but instead theoretically builds a model for future research. The article, which focuses on the lack of conceptual consideration of expatriate student mental health in the context of Middle East higher education, draws together the theories of Bronfenbrenner's Ecological Systems Theory, Lazarus and Folkman's Transactional Model of Stress and Coping, and Bandura's Social Cognitive Theory. The proposed framework suggests that psychological resilience may act as a mediator between sociocultural adaptation, psychological adjustment, perceived social support, and positive mental health. Self-efficacy as a belief-based personal resource is suggested as a potential moderator between psychosocial factors and resilient coping; that is, when students believe that they have the ability to cope with academic and social demands, they have the potential to transform the psychosocial resources into resilient coping. The article also points to the applicability of this construct in the UAE's multicultural higher education context and to other education issues of a general nature related to student wellbeing. The article offers a conceptual framework for future empirical studies that will be longitudinal, across levels and across cultures, and will clarify possible relationships between psychosocial resources, resilience, self-efficacy, and positive mental health. Moreover, it has implications for universities to create culturally responsive support structures for expatriate undergraduate students.

## Introduction

There has been a growing trend of internationalization in higher education in recent years, and an increasing number of students seek to study overseas. In the last 20 years, higher education enrolment has more than doubled globally (to around 269 million students in 2024), and international student mobility has tripled, with nearly 7.3 million students studying abroad. This expansion has sparked an increased interest in students' psychological, cultural, and social experiences studying in educational and cultural contexts different from their own. The United Arab Emirates is an especially pertinent context in this trend as it has set itself forth as a hub for Higher Education in the region and worldwide.

For instance, the number of students in the private higher education sector in Dubai has grown to 42,026 in 41 private higher education institutions for the academic year 2024–2025, of which 37 are international campuses. The number of international students in Dubai also rose by 29% year-on-year. However, the developments suggest that the UAE is not just a geography for international education but rather a fast-growing multicultural academic context where the psychosocial adaptation and mental wellbeing of international students as migrants should be studied more conceptually and empirically.

Expatriate students typically face many unique challenges while attempting to adjust and adapt to the different educational institutions, cultural norms, and social settings, as well as deal with psychosocial adjustment difficulties when trying to adjust/assimilate into a new social support network (Bethel et al., 2020; Nazir, 2024). Research consistently demonstrates that international students experience higher levels of psychological distress, anxiety, depression, and loneliness than domestic students (Misra et al., 2022; Yeboah et al., 2025). Globally, students from all over the world were negatively affected by the COVID-19 pandemic, resulting in increased levels of both academic and social isolation (Mosanya et al., 2021).

It is imperative to understand how psychosocial factors potentially impact a student's psychological health to develop effective psychosocial support and interventions. Studies have identified the positive influences of resilience, self-efficacy, and other protective coping strategies. Resiliency and self-efficacy have in reducing the effects of psychosocial stressors and positively impact student mental wellbeing (Chang et al., 2021; Mei et al., 2022; McDermott et al., 2020). The specific processes by which The specific processes through which these psychological factors influence outcomes, particularly within moderated mediation models, among expatriate student populations, are not well understood within a Middle Eastern context.

Whilst previous studies have looked at international student adjustment, social support, resilience, self-efficacy, and mental health, less attention has been paid to how these interact in contributing to positive mental health amongst the undergraduate international student population in Middle Eastern higher education settings. The UAE, in particular, is not represented in conceptual discussions on the interaction between sociocultural adaptation, psychological adjustment, perceived social support, resilience, and self-efficacy in a highly multicultural educational environment. The lack of a statistical model is not, therefore, the gap in this article, but rather the possible relationship between these constructs has not been sufficiently explained theoretically for the context of the UAE expatriate student. This article presents an explanatory conceptual framework, in which psychological resilience is assumed as a possible mediator between psychosocial factors and positive mental health, and self-efficacy is assumed as a moderator, as a personal resource that can strengthen the development of psychological resilience. This framework is mainly based on Bronfenbrenner's Ecological Systems Theory and incorporates the complementary perspectives of Lazarus and Folkman (1984) and Bandura (1986).

## Background and theoretical foundations

### Expatriate students in the UAE context

The United Arab Emirates has established a regional presence in higher education by attracting many expatriate students each year from a variety of cultures and countries. This multicultural setting provides both advantages and disadvantages for student adaptation and wellbeing. Recent research conducted in the UAE has reported a high number of mental health issues for university students, including increased levels of stress, anxiety, and depression, especially as a result of the COVID-19 pandemic (Misra et al., 2022). The study by Salgado et al. (2024) investigated the relationship between mental health and resilience among migrant students attending RAK Medical and Health Sciences University and found that, whilst most students possessed high positive mental health and average resilience, there was a statistically significant link between these two variables, suggesting that improving resilience is beneficial to the students' mental health. In addition, the varied cultural backgrounds of students enrolled in the UAE contribute to the diversity of cultural and social dynamics present in the UAE, which can impact student adaptation. Petkari et al. (2016) examined the relationship between character strengths and happiness and mental health among UAE youth in a multicultural context, providing further evidence of the importance of including cultural diversity when discussing wellbeing. Expatriate students will be expected to navigate through a multicultural environment that requires them to adjust academically, adjust socioculturally, and develop competency across cultures. To understand how students adapt to these processes, a general theoretical model should include all nine of the environments in which students exist.

## Core constructs

### Sociocultural adaptation

Sociocultural adaptation means the students' ability to adapt to the new cultural and social surroundings. For undergraduate students born abroad, this involves getting to know the norms of the host country, the expectations of the academic world, learning to communicate interculturally, and experiencing social situations in the university and other parts of society. In the UAE context, sociocultural adaptation is particularly relevant as students are exposed to students, teachers, and institutional culture and systems with a variety of cultural, linguistic, and religious backgrounds.

### Psychological adjustment

The emotional and cognitive adjustments of students to the demands of studying and living in a new environment are called psychological adjustment. It contains the capacity to handle homesickness, educational stress, identity negotiation, loneliness, uncertainty, and emotional stress linked to cross-cultural transition. Psychological adjustment is different from sociocultural adaptation because it is more a matter of emotional wellbeing than behavioral competence.

### Perceived social support

Perceived social support is the students' felt subjective perception of emotional, informational, and practical support provided by family, peers, faculty, university services, and the broader social network. Perceived support is drawn from co-national networks and relationships in the host country for expatriate students. Having a perception of support is as important as the actual support received, as it can have an impact on students' confidence, coping, and sense of belonging.

### Psychological resilience

Psychological resilience is the dynamic process of adapting, recovering, and functioning psychologically in response to a stressor, adversity, or major life transition. In this article, resilience is viewed as a coping adaptive process and not a static personality characteristic, and is nurtured through students' engagement with their social, school, and cultural contexts.

### Self-efficacy

Self-efficacy is the confidence of students in their capacity to plan and execute the activities that are necessary for the academic, social, and cultural demands. Some students with higher self-efficacy tend to perceive the difficulties or challenges as manageable, adopt a proactive coping style, seek support, and persevere in the face of difficulties. In the proposed framework, self-efficacy is considered a belief-based personal resource that has the potential to reinforce the link between the psychosocial resources and resilience.

### Positive mental health

Positive mental health is more than simply not suffering psychologically. Positive mental health comprises emotional wellbeing, life satisfaction, psychological functioning, purpose, social connectedness, and meaningful academic and social engagement. This strength-based understanding is particularly relevant for research with the expatriate student population, as it reflects a transition from a deficit-oriented perspective on stress, anxiety, and depression and a perspective that sees how students thrive in supportive educational environments.

## Theoretical framework

The original conceptual model is primarily based on the Ecological Systems Theory developed by Bronfenbrenner, along with two additional theoretical perspectives, which illustrate the specific mechanisms identified in the ecological framework. The hierarchical integration of these theoretical approaches adds breadth to understanding how psychosocial aspects interact with factors related to resiliency, self-efficacy, and mental health outcomes among expatriate students.

While psychological resilience and self-efficacy are conceptually similar constructs that share the common feature of protection, they are distinct. Psychological resilience is the adaptive ability or process whereby an individual will adapt or recover to a stressor and resume or recover psychological functioning. Thus, it has to do with adaptation in the face of adversity. Self-efficacy, on the other hand, involves people's self-confidence in their capacity to plan and perform the behaviors necessary to cope with a particular demand. The proposed framework considers resilience as a coping-based mechanism that can account for conversions of psychosocial resources to maintain positive mental health, and self-efficacy as a belief-based personal resource that can enhance or hinder conversion of psychosocial resources to resilience.

### Bronfenbrenner's ecological systems theory (primary framework)

The study was built on the Ecological Systems Theory proposed by Bronfenbrenner (1979, 1994) as the broad conceptual model or framework for understanding how multiple levels of nested environmental systems contribute to expatriate student mental health through a complicated set of relationships. It is proposed that human development and wellbeing develop in complex systems of relationships that are shaped by the multiple levels of the surrounding environment (Bronfenbrenner and Morris, 2006). In the case of expatriate students, their experience with these ecological systems occurs at five connected but distinct levels.

Microsystem: The microsystem represents the immediate environment where students interact face to face with the community, including their family and their relationships with peers, faculty, and roommates. Studies have shown that microsystem-level relationships, including peer relationships and faculty support, are significantly associated with mental health and adaptation among international students (an example is presented; Anandavalli et al., 2020; Celbis et al., 2025). The daily interactions within their university setting, dormitory, and immediate social network create the foundation of their microsystem in the UAE. The quality of the relationships and the support received via those relationships determine psychological wellbeing.

Mesosystem: The mesosystem represents the interactions that occur between two or more microsystems in the student's life. For expatriate students, this includes the relationship between their academic environment and their home life. between various groups of peers, and between student organizations and university support services. An example is found in the study by Zapata et al. (2025), which demonstrated that the interaction between family support (microsystem) and academic environment (mesosystem) contributes to the life satisfaction of university students and ultimately highlights the role that the relationship between two or more microsystems plays in the student's wellbeing.

Exosystem: The exosystem consists of external environmental settings where students are not participants, but which can influence their behavior. Expatriate students in the UAE are affected by policies and support services at the exosystem level, including academic policies, visa documents, and governmental education-related policies. Jackman et al. (2022) and Jackman et al. (2022) found that the policies of institutions, in addition to resource availability, at the exosystem level are related to doctoral students' mental health and wellbeing. Similarly, exosystem factors also shape how expatriate students adapt to their new environment (availability of mental health services, access to academic support programs, and the international office).

Macrosystem: The macrosystem is defined as the culture, including values, norms, and philosophies, that all societies and countries hold. For expatriate students studying in the UAE, the macrosystem consists of the value system and the attitude of the host country regarding international students and its national education plan, such as Education 33 (E33). Odriozola (2025) said that societal-level variables at the macrosystem level have a substantial impact on the mental health of university students; therefore, comprehensive intervention programs that address structural and cultural factors are necessary. The cultural diversity within the UAE, including cosmopolitan values and a variety of cultural communities, creates an entirely unique macrosystem that influences expatriate students' experiences.

Chronosystem: The chronosystem is the time dimension, incorporating life transitions, historical events, and development. As such, the chronosystem for expatriate students includes the transition from their home country to their host country and the progression through their programs and significant events such as the COVID-19 pandemic. Xu et al. (2024b) indicated that the wellbeing of doctoral students is influenced by many factors across all ecological systems and that time-related patterns/relationships between all aspects of life are all reflected in the chronosystem. The ecological systems framework is a particularly helpful model for understanding expatriate student mental health due to the recognition that the wellbeing of students is not based solely on individual characteristics but also, significantly, on the multi-level environmental contexts in which they are situated (Usher et al., 2019; Bernard et al., 2018). This view focuses on the interactions between the individual and their multiple environments rather than only the deficiencies of the individual; therefore, there is a need for multi-level intervention approaches that consider all ecological system levels (Joubert et al., 2024).

### Lazarus and Folkman's transactional model of stress and coping (supporting framework)

Lazarus and Folkman's (1984) Transactional Model of Stress and Coping is a theoretical construct that supports the concept of psychological resilience as a supportive mediating factor in the ecological systems framework. According to the model, stress is generated from the interactions between the person and the environment. This means that stress does not exist within the situation; rather, the extent to which someone perceives the situation as stressful depends on the resources they possess to cope with it (Buško et al., 2021; Zhao et al., 2023). There are two types of cognitive appraisal that occur simultaneously in an individual when confronted by an event or condition that is considered stressful.

Primary appraisal refers to the determination of whether the event is irrelevant, benign, or stressful (i.e., represents a loss/harm, a threat, or a challenge). Secondary appraisal relates to the determination of how to manage or cope with the stressful event. Psychological resilience is one of the primary coping resources available to people when coping with stress. Psychological resilience enables people to successfully cope with stress by using both a problem-focused coping strategy (addressing the source of stress) and an emotion-focused coping strategy (dealing with emotional reactions to the stressor). Research has demonstrated that the relationship between stressors and mental health outcomes exists in student populations through the mediating role of resilience. Chang et al. (2021) found that psychological resilience mediated the relationship between childhood trauma and depression in college students. In addition, Ho et al. (2022) found that resilience mediated the relationship between academic stress and depression in Vietnamese adolescents. These studies support the view of resilience as a dynamic process of coping that translates resources in the environment (i.e., ecological systems framework) into positive mental health outcomes.

The transactional model serves to complement the ecological systems framework by providing a psychological perspective on how students perceive and respond to the challenges that exist in the various ecological systems. The ecological systems framework identifies multiple contexts in the environment that impact student wellbeing, while the transactional model demonstrates active engagement by students with the demands of those environmental requirements through cognitive appraisal and coping processes.

### Bandura's social cognitive theory (supporting framework)

Social Cognitive Theory was developed by Bandura (1986) and provides a second supporting theoretical framework that explains the moderating role of self-efficacy within the ecological systems model. Social Cognitive Theory focuses on the importance of reciprocal determinism, or the interdependence of personal characteristics (such as cognition and beliefs), behavior, and environmental factors. As such, self-efficacy is a key personal characteristic that affects the way students interpret and respond to tasks in their environment and therefore influences the multiple psychological processes that contribute to the adaptation of expatriate students. For instance, self-efficacy has been found to influence goal-setting and aspiration levels; students with higher self-efficacy set more challenging goals and are more likely to persist in the face of obstacles and to experience less anxiety and greater emotional stability than students with lower self-efficacy.

In addition, self-efficacy influences the student's cognitive processing, including how they interpret feedback, as well as how they attribute their success or failure in completing a task (Schunk et al., 2020; Khuhro, 2024). Self-efficacy has been consistently demonstrated to moderate the relationship between environmental factors and mental health outcomes. In a longitudinal study involving students, Schönfeld et al. (2019) found that self-efficacy served as a mediator between daily stress and mental health across three different time points. In another study, Zhang et al. (2023) provided evidence of a bidirectional relationship between perceived social support and general levels of self-efficacy among graduate students. Chang (2025) identified self-efficacy as a mediator between physical activity and depression among Chinese students in Korea, and that social support moderated the relationship. Within the ecological systems framework, self-efficacy serves as a personal characteristic moderating the way that students convert environmental resources from multiple ecological systems into psychological resilience. Specifically, students who possess a higher level of self-efficacy are better able to convert environmental resources, such as social support and institutional resources, into psychological resilience, due to their confidence in their ability to manage the challenges associated with these resources. As such, the moderating role of self-efficacy addresses why students placed in similar environments (within the same ecological systems) will vary in the development of resilience, and, subsequently, their mental health.

### Conceptual framework and hypothesized relationships

A conceptual model which connects psychosocial factors with positive mental health in undergraduate expatriate students in the UAE is proposed in Figure 1. The model is conceptual and hasn't been tested empirically in the present article. The psychosocial factors of sociocultural adaptation, psychological adjustment and perceived social support are placed in a direct relationship with positive mental health. Psychological resilience is suggested as a mediator that is the connection between these psychosocial factors and positive mental health. Self-efficacy is suggested as a personal resource that can enhance the links between psychosocial and psychological resilience. In this context, students with high self-efficacy are more likely to use psychosocial resources to cope in a resilient way, whereas students with low self-efficacy are at greater risk of not benefiting from similar resources.

**Figure 1:**
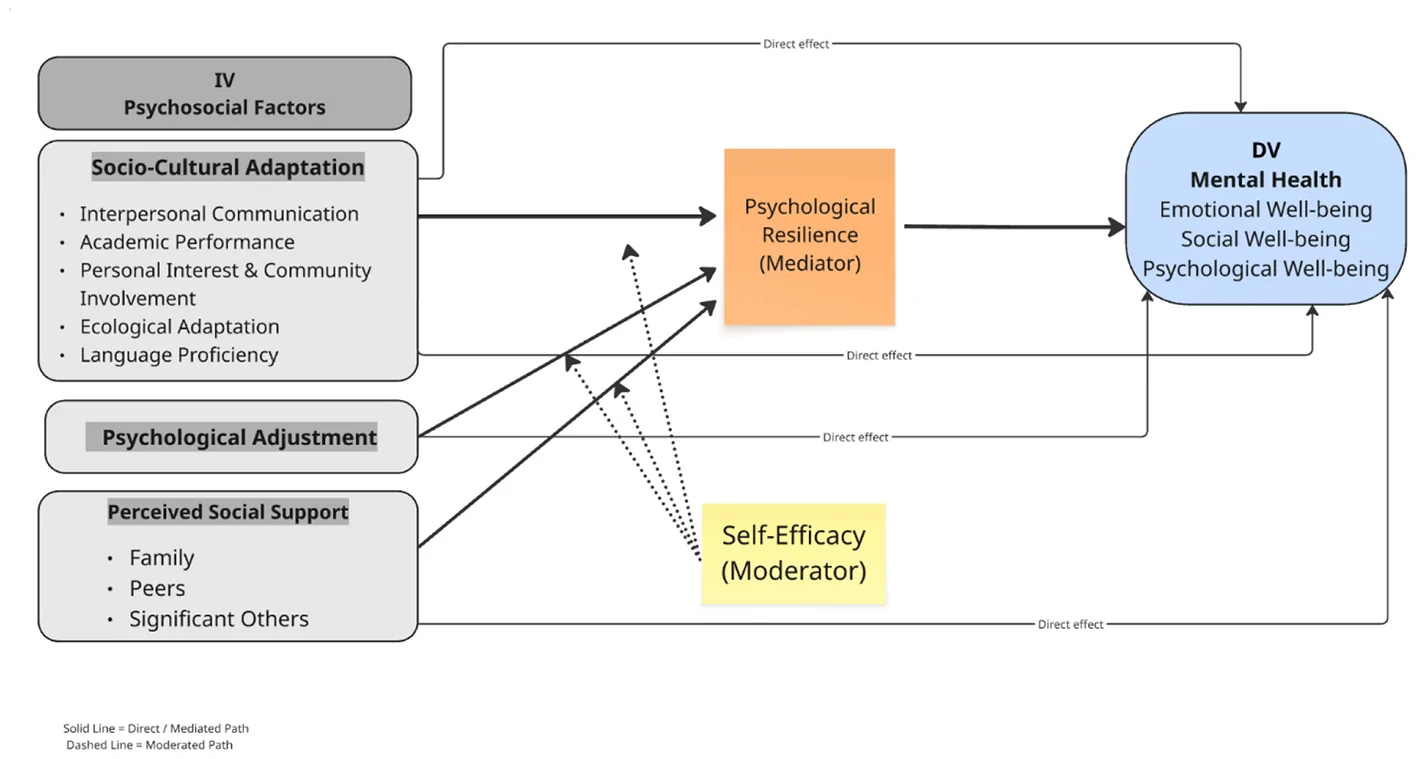
Proposed moderated mediation model. The model is conceptual and hasn't been tested empirically in the present article. Direct and mediated pathways are proposed by solid arrows. Direct paths linking psychosocial factors to positive mental health are proposed direct links. Psychological resilience is the proposed mediating mechanism, as illustrated by the pathway. The dashed arrow from self-efficacy is proposed to be a moderating path in the relationship between psychosocial factors and psychological resilience.

### Theoretical propositions

The following propositions are offered for future empirical study based on the literature reviewed and integrated theoretical framework:

P1: There is a positive relationship between sociocultural adaptation and positive mental health of undergraduate expatriate students in the UAE.
P2: Positive mental health is likely to be positively related to psychological adjustment in undergraduate students from abroad in the UAE.
P3: There is a positive relationship between perceived social support and positive mental health of undergraduate expatriate students in the UAE.
P4: Psychological resilience is suggested as a possible mediator between the level of sociocultural adaptation and positive mental health.
P5: Psychological resilience is suggested as a possible mediator between psychological adjustment and positive mental health.
P6: Psychological resilience is an intervening mechanism between perceived social support and positive mental health.
P7: It is suggested that the relationship between psychosocial factors and psychological resilience is dependent upon the Self-efficacy of the student; therefore, it is expected that the relationships will be stronger with students having higher levels of Self-efficacy.
P8: The indirect relationships between psychosocial factors and positive mental health via psychological resilience are anticipated to be stronger with a higher level of self-efficacy.

These propositions are not intended to be empirically substantiated relationships. Instead, they give a theoretically grounded foundation for future research that attempts to test this framework with appropriate quantitative, longitudinal, and multi-level designs.

### Future operationalization and empirical testing

The following propositions are offered for future empirical study based on the literature reviewed and integrated theoretical framework:

P1: There is a positive relationship between sociocultural adaptation and positive mental health of undergraduate expatriate students in the UAE.
P2: Positive mental health is likely to be positively related to psychological adjustment in undergraduate students from abroad in the UAE.
P3: There is a positive relationship between perceived social support and positive mental health of undergraduate expatriate students in the UAE.
P4: Psychological resilience is suggested as a possible mediator between the level of sociocultural adaptation and positive mental health.
P5: Psychological resilience is suggested as a possible mediator between psychological adjustment and positive mental health.
P6: Psychological resilience is an intervening mechanism between perceived social support and positive mental health.
P7: It is suggested that the relationship between psychosocial factors and psychological resilience is dependent upon the Self-efficacy of the student; therefore, it is expected that the relationships will be stronger with students having higher levels of Self-efficacy.
P8: The indirect relationships between psychosocial factors and positive mental health via psychological resilience are anticipated to be stronger with a higher level of self-efficacy.

These propositions are not intended to be empirically substantiated relationships. Instead, they give a theoretically grounded foundation for future research that attempts to test this framework with appropriate quantitative, longitudinal, and multi-level designs.

## Key variables: psychosocial factors as predictors

### Sociocultural adaptation

Sociocultural adaptation is the ability to effectively adapt to a new cultural setting and contains the competencies/skills necessary for an individual to successfully regulate their social interactions at the level of the new culture (i.e., the host environment). Sociocultural adaptation is an ecological dynamic and involves developing a person's ability to understand and adapt to the varying social norms, rules, and expectations across multiple ecological system levels (microsystem interpersonal relationships, mesosystem cultural differences between two or more cultures, and macrosystem's universal cultural expectations). Research shows that when an international student has difficulty with sociocultural adaptation, they experience greater psychological distress (see Bethel et al., 2020; Nazir, 2024); conversely, when successful socioculturally, they experience greater social integration, lower acculturative stress, and therefore improved psychological wellbeing. Jarrar et al. (2025) found that Nigerian students in Northern Cyprus who were successful with their acculturation experienced fewer mental health problems; additionally, participants who experienced acculturative stress also experienced higher levels of loneliness; social support had a moderating role in reducing the relationship between loneliness and mental health. Lari et al. (2025) supported the notion of sociocultural adaptation as being integral to the mental health of international students as a result of difficulties adapting due to the life transitions between ecological systems/contexts (i.e., difficulty socializing within new social groups). The proposed model suggests that sociocultural adaptation has both direct and indirect effects on positive mental health via psychological resilience (i.e., successful sociocultural adaptation results in the development of skills that build one's resilience, enabling a student to manage the subsequent challenge more effectively). In accordance with ecological theory, an individual's successful adaptation to one system level (e.g., microsystem) creates a capacity for adaptation across other system levels (e.g., mesosystem and macrosystem).

### Psychological adjustment

In the new environment, a person's emotional and cognitive responses to the demands of adaptation are actually part of that person's adjustment to the new environment, from an ecological perspective. The role of environmental systems on psychological adjustment refers to the way in which the psychological demands interact between several different environmental systems; e.g., adjusting to home by potentially feeling homesick from being separated from one's home microsystems; negotiating one's identity within new cultural macrosystems; and experiencing stress due to navigating unfamiliar institutional exosystems. International students' psychological adjustment is found to be central to their mental health outcomes (Jiang, 2018; Yeboah et al., 2025). As a result, when an international student does not achieve successful psychological adjustment, he/she typically will experience higher levels of depression, anxiety, and psychological distress. Conversely, when an international student achieves successful psychological adjustment, he/she will experience higher levels of positive mental health and life satisfaction. Buško et al. (2021) demonstrated that secondary/high school students' psychological adjustment during the COVID-19 pandemic was highly influenced by their subjective evaluations of online learning, consistent with the transactional model's affirmation of the importance of cognitive evaluation processes. The ecological perspective provides a conceptual framework that places psychological adjustment within the larger contexts of both the supportive and environmental demands experienced by the student from multiple environmental systems. The proposed model hypothesizes that psychological adjustment positively influences mental health through psychological resilience as a mediator. The psychological resilience developed due to an international student effectively meeting the demands associated with the cross-cultural transition will also provide the ability to protect him/herself against future experiences with stressors. This pathway of mediation fits with the transactional conceptualization of resilience, being viewed as a dynamic coping process that develops with successful adaptation to the demands of the environment.

### Perceived social support

Social support perceived is the individual's subjectively evaluated opinion of the social network targeted to provide support. When looking at the ecological systems perspective, social support takes place through many different levels or systems. At the microsystem level, for example, social support consists of emotional (friends), instrumental (family), and informational (faculty) support. At the mesosystem level, social support consists of relationships that connect different sources of support (for example, family and peer support). At the exosystem level, social support is provided by programs and services that provide formal structures to support. Multiple studies have consistently shown that social support is perceived as a strong predictor of mental health outcomes in university students (Holliman et al., 2022; Zhang et al., 2023). Additionally, research has shown that international students who have social support from co-nationals and from the host country are more likely to experience psychological wellbeing and successfully adapt (Bethel et al., 2020). Morris et al. (2020) illustrated how a multi-level social ecological framework showed how multi-level support systems enhance wellness for student veterans, supporting the premise of coordination among support across the ecological systems. The theory indicates that perceived social support has a direct and indirect effect on positive mental health through psychological resilience. Social support provides resources that help improve coping abilities through the development of resilience (Gong et al., 2022). The association between perceived social support and coping as an environmental factor demonstrates the transactional model view of social support as a critical resource for coping.

### Psychological resilience as mediator

Psychological resilience is the ability to adapt successfully to challenges, trauma, or major stressors. From the transactional model perspective, resilience is an adaptive process that plays a role in the coping process and is characterized by supporting characteristics and resources being mobilized to the individual in response to opposing demands from their environment. Further, resilience is not a static characteristic; instead, it emerges through ongoing transactions between the individual and their environment, which is congruent with the ecological systems framework's emphasis on person-environment interactions. Research indicates resilience mediates relationships between different stressors and mental health outcomes among student populations. For example, Chang et al. (2021) determined that psychological resilience mediated the relationship between childhood trauma and depression among college students. McDermott et al. (2020) demonstrated that resilience mediated the relationships between academic stressors and mental health outcomes in nursing students. Ho et al. (2022) found that resilience mediated the relationship between academic stress and depression in Vietnamese adolescents, and life satisfaction moderated this relationship. Xu et al. (2024b) examined psychological resilience and mediating relationships between negative emotions and post-traumatic growth among individuals affected by the COVID-19 pandemic.

The proposed model positions psychological resilience as a primary mediating mechanism through which psychosocial factors occurring across multiple ecological systems exert positive influence on mental health. Specifically, this model hypothesizes that:1) Sociocultural adaptation → Resilience → Positive mental health. Successful sociocultural adaptation from multiple ecological systems builds confidence and competency in one's abilities and positively influences resilience, impacting the ability to maintain positive mental health. (2) Psychological adjustment → Resilience → Positive mental health. Adjustment to the psychosocial demands associated with cultural transition enhances resiliency, protecting against psychological distress and promoting wellbeing. (3) Perceived social support → Resilience → Positive mental health. Social support from within multiple ecological systems creates resources that increase one's ability to cope with stressors or adversity and enhance resilience and promote positive mental health. There is empirical evidence to support that resilience mediates these relationships. For example, Salgado et al. (2024) determined that there is a statistically significant positive relationship between resilience and positive mental health among students studying in the United Arab Emirates. In addition, Celbis et al. (2025) identified adaptive resilience among doctoral students through their use of support from numerous ecological systems (microsystem support from their supervisors; mesosystem support from peer networks) during the COVID-19 pandemic. Overall, these studies provide support for understanding resilience as a critical link between environmental resources (in multiple ecological systems) and mental health outcomes.

### Self-efficacy as moderator

According to Bandura's Social Cognitive Theory, self-efficacy is one's belief in one's ability to plan and carry out actions needed to reach specific goals. The hypothesized model proposes self-efficacy as a moderator to strengthen the links between psychosocial factors and their association with psychological resilience. In so doing, it posits that the positive effect of social and cultural adaptation, psychological adjustment, and perceived social support on resilience is greater for high self-efficacy students than for low self-efficacy students. The theoretical rationale for this moderation hypothesis is found in the impact of self-efficacy on one's interpretation of and response to environmental demands across different ecological systems. It is expected that students with higher levels of self-efficacy will view difficulty and challenge as manageable, employ proactive coping strategies to overcome challenges, and persist in their efforts, leading them to be in a better position to convert environmental resources from the different ecological systems into psychological resilience. Self-efficacy's moderating role helps to explain why students have different levels of resilience when they experience the same environmental conditions. To support the claim that self-efficacy is a moderator, there are several empirical studies. Zhang et al. (2023) found a reciprocal relationship between perceived social support and general self-efficacy for postgraduate students. Chang (2025) noted that self-efficacy mediated the relationship between physical activity and depression in Chinese students enrolled in Korea, with social support as the moderator. Schönfeld et al. (2019) identified self-efficacy as a means by which daily stress affected mental health among students at three time points. Mei et al. (2022) determined that self-efficacy was inversely related to psychological distress for nursing students and that resilience had a protective effect. Self-efficacy, in relation to an ecological systems framework, is a variable that embodies a personal characteristic that moderates the ability of students to use resources from multiple ecological systems. For example, high self-efficacy students are expected to be more successful in seeking and using social support from their microsystem relationships, accessing institutional resources at the exosystem level, and adapting to cultural norms/pressures at the macrosystem level. The ability to mobilize environmental resources leads to greater psychological resilience.

### Positive mental health as outcome

The model proposed for research on positive mental health as the primary outcome measure represents a paradigm shift from deficit-based models that are focused on psychopathology (the absence of mental health) to strength-based models that focus on wellness and flourishing. Positive mental health includes emotional wellbeing (positive affect and satisfaction with life), psychological wellbeing (autonomy, personal growth, purpose in life), and social wellbeing (social integration and contributions). This understanding of positive mental health connects to the ecological systems framework's view of optimal development of individuals and wellbeing being determined by a supportive relationship between the individual and their environment. Research has become more aware of the importance of studying positive mental health outcomes, not only the absence of mental illness. For example, Salgado et al. (2024) studied the positive mental health of migrant students in the UAE using the Positive Mental Health Scale, demonstrating that the majority of students possess high levels of positive mental health. Holliman et al. (2022) researched several dimensions of psychological wellbeing of Chinese international students in the UK, including flourishing, life satisfaction, and psychological distress; demonstrating that adaptation and social support can independently influence the development of these outcomes. The perspective of the ecological systems framework supports that positive mental health is the result of successful adaptation across numerous environmental systems. When students experience supportive relationships in their microsystem, connectedness with their mesosystem, resource availability in their exosystem, and validation through their macrosystem, they are more likely to develop positive mental health. The proposed research will examine the potential direct and indirect influence of psychosocial factors (from all ecological systems) on positive mental health through psychological resilience and the influence of self-efficacy on these relationships.

### Integration of theoretical perspectives

The proposed moderation meditation model offers theoretical integration by establishing Bronfenbrenner's ecological theory of systems as the primary framework for understanding how many layers of nested environmental systems influence the mental health of students. Two other supporting theories offer more specific explanations of the mechanisms in the ecological model. Integrating these three frameworks in a hierarchical manner will yield a more comprehensive, simplified explanation of how psychosocial factors affect the mental health of expatriate students. The primary theoretical base of Bronfenbrenner's ecological theory serves as the main framework for understanding the influence of multiple nested environmental systems on student mental health. The theory places emphasis on the interaction between persons and the environment across many levels (microsystem, mesosystem, exosystem, macrosystem, and chronosystem), creating a comprehensive lens through which the psychosocial factors of the model are construed. Sociocultural adaptation, psychological adjustment, and perceived social support are not simply independent characteristics of students, but are developed through the students' interactions within or with the different levels of nested environmental systems.

The ecological framework of Bronfenbrenner's theory explains how and where psychosocial factors operate consistently within immediate and close networks of people (microsystems), the connections between different contexts or settings (mesosystems), institutional structures (exosystems), and in large-scale or cultural systems in the world (macrosystems). The transactional model of stress and coping by Lazarus and Folkman functions as a supporting theoretical framework that will explain how student psychological resilience (as operationalized through the active coping of students) is the mediator between psychosocial factors and the mental health of students. While the ecological framework uses multiple nested environmental systems to explain the influences on student wellbeing, the transactional model describes how students actively interpret and respond to demands placed on them by those nested systems. Resilience explains how actively coping with environmental demands by utilizing cognitive appraisal (primary and secondary appraisal) and coping strategies (problem-focused and emotionally-focused) will yield students a positive adaptation. Therefore, resilience serves as the mediator between psychosocial factors and student mental health as it describes active coping through which students convert environmental resources into positive adaptation.

One additional supporting framework, Bandura's social cognitive theory, describes how self-efficacy serves as a moderator of the ecological framework of systems that impact student mental health. The ecological framework identifies the environmental resources in the nested systems, and the transactional model of stress and coping describes how students respond to environmental demands; accordingly, Bandura's social cognitive theory explains why students respond differently to the same environmental conditions. Self-efficacy is a belief system that affects how students perceive environmental demands, how students establish goals, how much effort students expend, and whether students will continue to work through difficulty. Students with higher levels of self-efficacy will be better positioned to use the environmental resources from each of the nested systems and to utilize effective coping as defined by the transactional model of stress and coping, and, ultimately, develop resilience.

The hierarchical integration of the proposed three theoretical perspectives for understanding the mental health of expatriate students provides a number of possible benefits. First, it specifies the established primary framework (i.e., ecological systems theory), which serves as the complete basis for understanding the mental health of expatriate students. Second, the three supporting theoretical frameworks collectively explain specific mechanisms (i.e., mediation of resilience and moderation of self-efficacy) without contributing to theoretical overlap. Third, the integration of perspectives acknowledges that mental health is a function of multiple interacting factors at all levels of analysis-from individual cognition and beliefs (social cognitive theory), to dynamic coping processes (transactional model), and to multiple nested environmental systems (ecological systems theory). Fourth, the integration of theoretical perspectives provides direct access to multiple leverage points and provides guidance for creating interventions delivered across the interaction of multiple ecological systems, coping processes, and self-efficacy beliefs. The integration of the three theoretical perspectives provides a very viable theoretical framework for understanding expatriate student mental health in the multicultural setting of the United Arab Emirates. The ecological framework portrays the complicated navigation of several cultural systems by the expatriate student; the transactional model illustrates the active coping processes of expatriate students adjusting to cross-cultural demands, and the social cognitive theory identifies individual differences in the ability to adapt. Together, the three theoretical perspectives will provide a strong foundation for understanding the current complexities of how psychosocial factors impact the mental health of expatriate student populations.

## Implications for research and practice

### Research implications

This proposed conceptual framework is based on the ecological systems theory, as well as supported by other perspectives, including transactional and social-cognitive, and will provide a research basis for future empirical evidence regarding expatriate student mental health in the United Arab Emirates (UAE) and other similar locations of expatriates throughout the Middle East. The following highlighted areas are possible directions for future research based on this proposed framework.

### Empirical testing of the moderated mediation model

Future empirical studies can start with a cross-sectional survey design to investigate relationships between the proposed constructs, but cross-sectional designs have drawbacks in studying temporal processes, developmental change, and directionality. A longitudinal design would be more suitable for studying the development of expatriate students' sociocultural adaptation, psychological adjustment, perceived social support, resilience, self-efficacy, and positive mental health in Ecological Systems Theory, which takes the chronosystem into account. Future research includes data collection at a number of stages: before arrival, after one semester, after one year, and in further years. These would enable investigations into the relationships between psychosocial resources and subsequent resilience and positive mental health, between mental health and subsequent adaptation, and between changes in self-efficacy with experience in the educational environment of the new country.

### Multi-level ecological assessment

The development and validation of assessments that include variables from each level of ecology will require research. Microsystem-assessed variables include the quality of peer relationships and faculty support; mesosystem-assessed variables assess how well the various support systems are coordinated with one another; exosystem-assessed variables include the effects of institutional policies; and macrosystem-assessed variables include the cultural environment as well as societal attitudes. Conducting multiple levels of assessment results in a better understanding of how the various ecological factors affect expatriate students' mental health (Usher et al., 2019; Odriozola, 2025).

### Cross-cultural comparisons

Research should look into whether the proposed model applies to a range of expatriate student cultures in the UAE. The varied nature of the UAE's student body allows for comparative studies, making it possible to examine cultural differences between psychosocial factors, resilience, self-efficacy, and mental health outcomes. Also, this research looks at how cultural values on macrosystem levels shape the various ecological adaptation processes as discussed by Petkari et al. (2016).

### Longitudinal studies of adaptation trajectories

Ecological systems theory recognizes the significance of time in an individual's environment and interactions with their surroundings through a theoretical lens referred to as the chronosystem. With regard to longitudinal studies, it is important to evaluate the evolution of the adaptation trajectory for international students over time to identify important transition points and the impact that previous adaptation experiences have on subsequent outcomes to help determine when and how to implement support interventions (Xu et al., 2024a).

### Intervention studies

The framework provides numerous possibilities for intervening at different ecological levels. To assess intervention effectiveness targeting microsystems (e.g., peer support programs), mesosystems (e.g., coordination of academic and social support systems), exosystems (e.g., organizational policy changes), and individual factors (e.g., resilience training, building self-efficacy), randomized controlled trials are recommended. Implementing multi-level interventions that target across multiple ecological levels results in greater success (Anandavalli et al., 2020).

## Practice implications

The new framework is primarily based on Ecological Systems Theory, and outlines many important ways that higher education can have an impact on students through educational practice and support services.

### Multi-level support systems

Higher education institutions must offer comprehensive multi-level support programs, addressing different supporting factors across multiple ecological systems. At the microsystem level, support programs include the establishment of positive peer support systems, mentoring programs, and positive student-faculty interactions. At the mesosystem level, support services will be able to coordinate their services (i.e., academic advising, counseling, and international student offices) for integrated support to the student. At the exosystem level, higher education institutions will need to put in place supportive institutional policies and accessible supportive services. At the macrosystem level, higher education institutions will create an inclusive campus environment with a focus on valuing diversity (Jackman et al., 2022).

### Ecological assessment and intervention planning

Ecological assessment methods should be adopted by student support services to measure and evaluate the students' experience throughout the various layers of each system. Each assessment should evaluate the strengths and challenges that occur both at the level of the microsystem through relationships, mesosystem through connections to the individual within the exosystem, and at the structural or cultural level of the macrosystem. Intervention plans can then be created to address the specific levels within an ecological system where students are experiencing barriers to success (Joubert et al., 2024).

### Resilience-building interventions

As psychological resilience is the key mediator in this model, universities should provide programs that promote resilience based on scientific evidence. These programs should support students with both problem-focused coping (dealing directly with the source of stress) and emotion-focused coping (managing how they deal with their feelings about what they are experiencing). Additionally, the programs will teach students how to accurately evaluate the stress they encounter and find the proper coping strategies (Mosanya et al., 2021; Xu et al., 2024b).

### Self-efficacy enhancement

According to the moderating factor, improving students' confidence in their capacity to deal with academic and social challenges is the basis for designing intervention programs. In line with Social Cognitive Theory, the four main means through which self-efficacy is built or increased are: (1) Mastery Experiences, providing opportunities for learners/successes. (2) Vicarious Experiences, learning from observing how others have experienced success through their learning, and established methods of reinforcement. (3) Verbal Persuasion, through verbal reinforcement, i.e., constructive feedback, positive reinforcement, etc. (4) Physiological/Emotional State Management, reducing anxiety. Intervention programs need to focus on developing both academic self-efficacy and social self-efficacy (Jiang, 2018; Zhang et al., 2023).

### Sociocultural adaptation support

Universities are thought to create structured programs that assist expatriate students with the sociocultural adaptation process and develop skills to function within the host culture. Program content should cover several ecological levels of adaptation, e.g., from practical skills for daily socializing (microsystem), to understanding customs and norms of the broader culture (macrosystem), to include how these two cultures intersect (mesosystem) (Bethel et al., 2020).

### Social support network development

Developing social networks at universities is necessary across various ecological systems. Examples of these include creating peer support networks (microsystem), connecting academic and social environments (mesosystem), and creating more formalized institutional support services (exosystem). Programs developed should focus on helping students build their co-national support systems and friendships in their new host nations, as both help improve wellbeing (Holliman et al., 2022).

### Early identification and ecological intervention

Support services should be using screening methods to discover how well the student is doing in many aspects of their multiple ecological systems during the early stages of their academic careers. Once a challenge has been identified, interventions will address the particular level of the ecological system where the student is experiencing the challenge(s). For example, if a student is experiencing difficulty in his/her peer relationships (microsystem), the student benefits from social skills training and/or peer connection programs. If a student experiences barriers (exosystem) that require advocacy and/or institutional policy changes (Misra et al., 2022).

## Policy implications

The framework of ecological systems coordinates with national educational policy and supports the objective of Education 33 (E33) to create the UAE as a global educational center of excellence. Therefore, it is recommended that to culturally re-establish the next generation of UAE citizens, the implementation of the following four policy initiatives will correlate the mental health and wellbeing of UAE's students to a successful learning experience:

### National mental health strategy

To ensure that higher education is meeting the needs of students mentally through developing a comprehensive national mental health strategy, which will address specific multiple ecological systems that impact student mental health through providing guidelines from institutional support services to promote an inclusive campus culture and provide collaboration between various support systems that operate as one (i.e., exosystem, macrosystem, and mesosystem).

### Quality standardization for multi-level support

To ensure that all universities within the UAE have the required resources in place to adequately support their students physically, financially, and mentally, through establishing a comprehensive quality standardization policy for all campuses, from direct support (microsystem/exosystem) to promoting an inclusive campus environment through providing the appropriate services and policies that positively impact campus climate (macrosystem).

### Funding research on the expatriate student's mental health using an ecological systems framework

To establish a fund to promote research that examines and evaluates the mental health status of expatriate students through research that applies the ecological systems framework to support the development of effective evidence-based interventions based on the collected data from their families and/or homes through each of the multiple ecological systems.

### Institutional accountability for student mental health and wellbeing through the evaluation of the ecological system

To establish an assessment system for the universities to monitor and evaluate student mental health status through applicable established assessment methodologies, and combined with creating inclusive environments, promote the mental wellbeing of the students.

### Contextual and cultural limitations of the proposed framework

The suggested framework should be flexible and cognizant of the diversity of expatriate undergraduate students in the UAE. Not all expatriate students are alike. These variations can include a difference in nationality, language background, religion, socioeconomic status, migration history, and visa status, family presence in the UAE, prior international exposure, and cultural proximity to the host society. These differences could influence the nature of sociocultural adaptation, psychological adjustment, perceived social support, self-efficacy, resilience, and positive mental health for students. For instance, students with a culturally or linguistically close background find their way into the social sphere more easily than students with a more distant cultural and linguistic background. Likewise, the extent to which pupils have access to academic, psychological, and social support is greater depending on the financial resources or family support they have.

The proposed framework will be different depending on institutional differences. Students living in an international branch campus, public, private, or specialized institutions have different academic expectations and support systems, counseling services, and peer networks. Disciplinary cultures also can make a difference, as students in the health sciences, engineering, business, education, or humanities encounter varying workloads, assessment methods, professional expectations, and stresses. The differences indicate that the proposed relationships may not be consistent among all expatriate student groups in the UAE. It would be important for future research to investigate whether the proposed framework is different for student subgroups, different institutional types, disciplines, socioeconomic and cultural communities.

## Conceptual limitations

There are a number of conceptual constraints to be acknowledged. First, the suggested framework is theoretical and not tested in the present article. Thus, the suggested pathways are best considered to be a theoretical suggestion rather than established relationships. Second, the relation between psychosocial adaptation, resilience, self-efficacy, and positive mental health is reciprocal and dynamic, as the framework suggests some directions. Third, Ecological Systems Theory is difficult to translate into measurable constructs due to the complex, overlapping, and context-dependent nature of ecological influences. For instance, peer support could take place at the microsystem level, and it is influenced by institutional policies, cultural norms, and social expectations. Fourth, expatriate undergraduate students in the UAE are extremely varied, with the framework potentially not being relevant to all nationalities, socioeconomic statuses, institution types, or majors. Lastly, the model only considers selected psychosocial resources, and other factors like language barriers, discrimination, financial stress, academic workload, and visa insecurity, as well as other factors like religious identity, family expectations, and access to mental health services, are not fully explained.

## Conclusion

This conceptual paper aimed to present a theoretically based moderated mediation model to explain the phenomenon of positive mental health in UAE expatriate undergraduate students. The article does not provide any empirical results, but rather a model for explanation as a guide for future research on how psychosocial factors are connected to positive mental health in a multicultural context in higher education. The framework largely draws on Bronfenbrenner's Ecological Systems Theory, which emphasizes the importance of various environmental systems for students' wellbeing. This ecological perspective is aided by Lazarus and Folkman's Transactional Model of Stress and Coping, which explains their proposed role of psychological resilience as a coping-related mechanism, and Bandura's Social Cognitive Theory, which helps explain their proposed moderation of self-efficacy.

Based on the model, sociocultural adaptation, psychological adjustment, and perceived social support are indirectly related to positive mental health by psychological resilience, as well as directly related to positive mental health. Self-efficacy is suggested as a personal belief resource that could enhance students' personal capacity to transform psychosocial resources into resilience. The framework helps clarify the conceptual roles of the two protective constructs: resilience and self-efficacy, which are differentiated as adaptive coping and a belief in one's ability to cope with demands, respectively.

The article makes a key contribution to the literature on the mental health of expatriate students by moving the focus from an individual level of distress to a psychosocial and ecological level that helps to support good mental health. This is particularly pertinent in the UAE, where the internationalization, cultural diversity, and growth of international campuses of higher education are rapidly progressing. The framework is also of practical use for universities in that it highlights that a focus on student wellbeing can only be achieved by looking beyond individual counseling or response to crisis. Rather, multi-level supports are encouraged to be considered that support students' peer relationships, faculty engagement, social support, culturally responsive services, resilience building, and academic and social self-efficacy.

The framework is to be read with care. The proposed pathways are theoretical and need to be tested through empirical research. The suggested relationships are for future studies to be conducted in a longitudinal and cross-cultural design to see whether the relationships are the same over time and across student groups, institutional contexts, and disciplinary contexts. The relationships between positive mental health and psychosocial adaptation, between positive mental health and resilience, and between resilience and self-efficacy should also be considered by researchers. This article presents a theoretically integrated but empirically testable framework that can serve as a starting point for future research and practice to promote wellbeing and success for the expatriate undergraduate students in the UAE.

## Data Availability

No datasets are included in this manuscript or supplementary material. This study is conceptual in nature and is based entirely on previously published literature. Therefore, no primary or secondary datasets were generated, analyzed, or deposited in any repository.
